# Efficient Coding and Energy Efficiency Are Promoted by Balanced Excitatory and Inhibitory Synaptic Currents in Neuronal Network

**DOI:** 10.3389/fncel.2018.00123

**Published:** 2018-05-03

**Authors:** Lianchun Yu, Zhou Shen, Chen Wang, Yuguo Yu

**Affiliations:** ^1^Institute of Theoretical Physics, Lanzhou University, Lanzhou, China; ^2^The School of Nationalities' Educators, Qinghai Normal University, Xining, China; ^3^Cuiying Honors College, Lanzhou University, Lanzhou, China; ^4^Department of Physical Science and Technology, Lanzhou University, Lanzhou, China; ^5^State Key Laboratory of Medical Neurobiology, School of Life Science and Human Phenome Institute, Institutes of Brain Science, Center for Computational Systems Biology, Fudan University, Shanghai, China

**Keywords:** neuronal network, energy efficiency, excitation/inhibition ratio, mutual information, bistable neuron

## Abstract

Selective pressure may drive neural systems to process as much information as possible with the lowest energy cost. Recent experiment evidence revealed that the ratio between synaptic excitation and inhibition (E/I) in local cortex is generally maintained at a certain value which may influence the efficiency of energy consumption and information transmission of neural networks. To understand this issue deeply, we constructed a typical recurrent Hodgkin-Huxley network model and studied the general principles that governs the relationship among the E/I synaptic current ratio, the energy cost and total amount of information transmission. We observed in such a network that there exists an optimal E/I synaptic current ratio in the network by which the information transmission achieves the maximum with relatively low energy cost. The coding energy efficiency which is defined as the mutual information divided by the energy cost, achieved the maximum with the balanced synaptic current. Although background noise degrades information transmission and imposes an additional energy cost, we find an optimal noise intensity that yields the largest information transmission and energy efficiency at this optimal E/I synaptic transmission ratio. The maximization of energy efficiency also requires a certain part of energy cost associated with spontaneous spiking and synaptic activities. We further proved this finding with analytical solution based on the response function of bistable neurons, and demonstrated that optimal net synaptic currents are capable of maximizing both the mutual information and energy efficiency. These results revealed that the development of E/I synaptic current balance could lead a cortical network to operate at a highly efficient information transmission rate at a relatively low energy cost. The generality of neuronal models and the recurrent network configuration used here suggest that the existence of an optimal E/I cell ratio for highly efficient energy costs and information maximization is a potential principle for cortical circuit networks.

**Summary:**

We conducted numerical simulations and mathematical analysis to examine the energy efficiency of neural information transmission in a recurrent network as a function of the ratio of excitatory and inhibitory synaptic connections. We obtained a general solution showing that there exists an optimal E/I synaptic ratio in a recurrent network at which the information transmission as well as the energy efficiency of this network achieves a global maximum. These results reflect general mechanisms for sensory coding processes, which may give insight into the energy efficiency of neural communication and coding.

## Introduction

Through evolution, the morphology, physiology, and behavior of animals' organs are shaped by selective pressures that act to increase the ratio of benefits accrued to costs incurred to ensure fitness for survival. Human brains are composed of tens of billions (10^11^) of neurons and several hundred trillion synaptic connections that process and exchange prodigious amounts of information efficiently over a spatially distributed neural network on a timescale of milliseconds (Rousselet et al., 2004). These information processes require huge amounts of energy, approximately 20% of the energy used by the entire body, although they occupy only 2% of the body mass (Clarke and Sokoloff, 1999). This suggests that our brains are required to operate energy efficiently so that the brain process as much information as possible at the lowest energy cost (Barlow, 1961; Levy and Baxter, 1996). As a selective pressure, the demand for energy efficiency could be sufficiently large to evolutionarily affect the design of the brain (Niven and Laughlin, 2008).

In previous decades, substantial advances have been made to determine the strategy used by neural systems to work efficiently while saving energy, including optimizing ion channel kinetics (Alle et al., 2009; Schmidt-Hieber and Bischofberger, 2010), developing a warm body temperature to minimize the energy cost of single action potentials (Yu et al., 2012), optimizing the number of channels on single neurons and the number of neurons in neuronal networks (Schreiber et al., 2002; Yu and Liu, 2014; Yu et al., 2016), maintaining a low probability of releasing neurotransmitters at synapses (Levy and Baxter, 2002; Harris et al., 2012), representing information with sparse spikes (Olshausen and Field, 2004; Lorincz et al., 2012; Yu et al., 2014), optimizing the inter- and intra-regional wiring of the cortex (Mitchison, 1991; Chklovskii and Koulakov, 2004), arranging functional connectivity among brain regions in the form of a “small world” network (Bassett and Bullmore, 2006; Tomasi et al., 2013), and other techniques. These studies demonstrate the possibility that a trade-off between energy cost and information processing capacity driven by selective pressure could shape the morphology and physiology of neural systems to optimize for energy efficiency.

Cortical information processing accounts for a considerable proportion of the brain's energy consumption (Attwell and Laughlin, 2001; Lennie, 2003; Howarth et al., 2012), and a large fraction of this energy is consumed by action potentials, which are electrical signals and rely on the potential energy stored in transmembrane ion gradients (Attwell and Laughlin, 2001). The cortex's restricted energy budget places limits on the mean spike rate and hence on neural processing, suggesting that the cortex may be under strong selective pressure to save energy and increase efficiency (Laughlin, 2001; Hasenstaub et al., 2010; Yu et al., 2012). Although the factors listed above, such as action potentials with minimal energy cost, the size of the systems involved, and optimized wiring length, could substantially contribute to energy efficiency in the cortex, it is not clear whether and how cortical neural systems could achieve maximal energy efficiency, especially when their particular characteristics are considered.

One such characteristic of the cortex is its conservation of the overall ratio of excitatory to inhibitory neurons, where most neocortical neurons (70–80%) are excitatory pyramidal neurons; the remainders (20–30%) are inhibitory interneurons (DeFelipe et al., 2002; Markram et al., 2004). This conserved ratio is proportional to certain ratio between the excitational and inhibitory (E/I) synaptic currents as measured in experiments (Shadlen and Newsome, 1994, 1998; Somogyi et al., 1998). The certain E/I synaptic current ratio is time-variable depending on precise time scales. It causes the membrane potential to fluctuate slightly below the spiking threshold, generating spike trains with highly variable inter-spike intervals, in agreement with extracellular recordings from single cortical neurons (van Vreeswijk and Sompolinsky, 1996; Shadlen and Newsome, 1998; Somogyi et al., 1998). Balanced or unbalanced synaptic currents may vary the membrane time constant to increase temporal resolution and extend bandwidth (Bernander et al., 1991; Paré et al., 1998; Destexhe et al., 2003; Mittmann et al., 2005), alter the sensitivity and functionality of the neuron by changing its gain (Wehr and Zador, 2003; Wilent and Contreras, 2005; Wolfart et al., 2005; Rudolph et al., 2007), and provide rich repertoire of states, including synchronous and asynchronous firings (Brunel, 2000; Renart et al., 2010). Large E/I ratio is observed to increase correlations in spikes, thereby decreasing independent coding components. A small E/I ratio may also reduce coding information, because of drop in the overall level of neural activity. Experiments on cortical cultures, anesthetized rats, awake monkeys, and computer models show that cortical entropy and information transmission are maximized for an intermediate E/I ratio, at which ongoing activity emerges as neuronal avalanches (Shew et al., 2011). Recently, Yang et al. demonstrated that multiple experimentally observed cortical activities such as irregular firing, synchronized oscillations and neural avalanches co-emerge simultaneously in the E-I balanced neuronal networks (Yang et al., 2017). Therefore, the E/I ratio affects many aspects of information processing in cortical neuronal systems(Deco et al., 2014; Denève and Machens, 2016).

By simulating single Hodgkin-Huxley (HH) neurons receiving both excitatory and inhibitory inputs, Sengupta et al. found that balanced synaptic currents evoke fewer spikes per second, but spikes evoked by balanced synaptic inputs are more informative (bits/spike). Thus, both coding efficiency and energy efficiency are promoted at the level of a single neuron with balanced synaptic current inputs (Sengupta et al., 2013). Later, Yang et al. demonstrated in the E-I balanced network that with the co-emergence of multiple cortical activities, the network achieves maximal energy efficiency and minimal energy cost, when neuronal firings are shaped by moderate synchrony to reduce redundant spikes, and the critical dynamics with neuronal avalanches takes place (Yang et al., 2017). However, whether energy efficiency could be maximized by the E/I cell ratio and balanced synaptic currents in a neuronal network in the context of population coding is still unknown. In this study, we simulated a neuronal network composed of HH neurons in a noisy environment and studied the effects of the balanced E/I synaptic currents on the information transmission and energy efficiency of this network in response to pulse-like inputs. Though the input information may be carried by the inter-spike intervals in the time coding scheme, to simplify the issue, in this paper we only consider the information encoded by the firing rate patterns of the neurons, while temporarily ignore the potential information carried by the inter-spike intervals. Then we measured the information transmission of the network with Shannon's information entropy theory, which quantify information as the amount of disorder or uncertainty in the firing patterns (Shannon, 2001). We simultaneously calculated the changing of energy expenditure along with the E/I synaptic current ratio of this network in response to the inputs. Afterwards, the energy efficiency of the network is measured as the ratio of mutual information between firings patterns generated by the network and the input pulse strength to its energy expenditure. We found that both information transmission and energy efficiency are maximized by the optimal E/I synaptic current ratio. We also investigated how the background noise intensity, fixed energy cost, and ratio of synaptic energy cost to action potential energy cost affect the energy efficiency of the network. By incorporating the effects of excitatory and inhibitory synaptic currents as a net synaptic current, we modified the response function of a bistable neuronal model that we developed in previous work (Yu and Liu, 2014; Yu et al., 2016) to obtain an analytical solution for the mutual information and energy efficiency of the network. We then demonstrated that optimal net synaptic currents are capable of maximizing both the mutual information and energy efficiency.

This study is organized as follows: The network model and the methods involved in the calculation of the mutual information and energy cost are presented in section Model and Method, along with the mean field approximation solution for bistable neuron model incorporated with net synaptic currents; In section Results, we describe the response of the network to pulse inputs and the dependence of the mutual information and energy cost on the E/I cell ratio. We also demonstrate in this section the effects of noise intensity, fixed energy cost, and the ratio of synaptic energy cost to action potential energy cost. Finally, we present analytical results from the bistable neuronal models to demonstrate the possibility of maximizing energy efficiency with net synaptic currents. Section Discussion is devoted to discussion, and conclusions are given in section Conclusion.

## Model and method

### Neuronal network model

**Figure 1:**
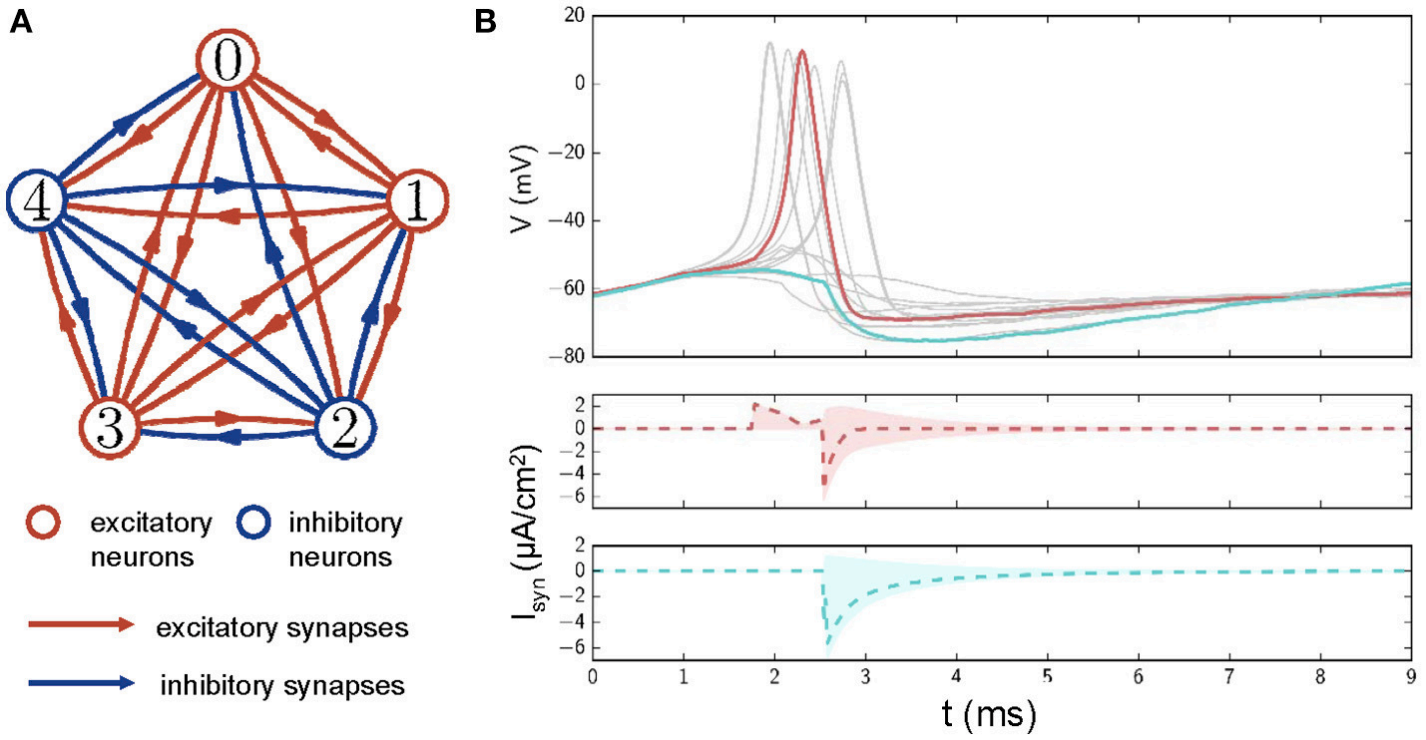
**(A)** A diagram of a neuronal network with excitatory and inhibitory synaptic couplings. Red circles represent excitatory neurons, and blue circles represent inhibitory neurons. Lines with arrows mark the connections and directions of synapses. Red: excitatory connections; Blue: inhibitory connections. **(B)** Top: Examples of membrane potential traces for neurons in the network after stimuli are applied at 0 *ms*. To have a better view, only membrane potential traces of 15 neurons are displayed. Middle: synaptic currents for the neuron whose membrane potential is shown in red. Dashed lines represent the averaged synaptic currents from the other neurons. The red shadow represents the range covered by synaptic currents from other neurons. Bottom: as in the middle plot, these traces represent a neuronal membrane potential in cyan. Δ*I* = 6.05μ*A*/*cm*^2^ and *D* = 0.1 in these simulations.

To investigate the effects of E/I cell ratio on the information transmission and energy consumption of neural systems, we first constructed a neuronal network with N neurons that were all-to-all connected with each other through excitatory and inhibitory synaptic connections (Figure 1A). The membrane potential dynamics for the i-th neuron (i = 1, 2,…,N) in the network is described by the classical HH model with Gaussian white noise:

(1) $$\begin{array}{l} C\frac{dV_{i}}{dt}=-g_{Na}m_{i}{}^{3}h_{i}(V_{i}-E_{Na})-g_{K}n_{i}{}^{4}(V_{i}-E_{K})-g_{L}(V_{i}-E_{L})\\ \;+I_{Syn}^{i}(t)+\xi_{i}(t)+I(t), \end{array}$$

where C is the membrane capacitance, and *V*^*i*^ is the membrane potential. *g*_*Na*_, *g*_*K*_, and *g*_*L*_ are maximal conductance per unit area for each kind of channel, respectively. *E*_*Na*_, *E*_*K*_, and *E*_*L*_ are reversal potentials of the sodium, potassium and leakage currents, respectively. The gating variables m, n, and h obey the following equations:

(2) $$\frac{dx_{i}}{dt}=\alpha_{x_{i}}(V_{i})(1-x_{i})-\beta_{x_{i}}(V_{i})x_{i},\;x_{i}=m_{i},n_{i},h_{i}$$

where α_*x*_*i*__(*V*_*i*_) and β_*x*_*i*__(*V*_*i*_) are voltage-dependent opening and closing rate functions of the ion channels. These rate functions are given in the Table 1, along with other parameters used in the simulation. In Eq. (1), ξ_*i*_(*t*) is the background Gaussian white noise with $\langle \xi_{i}(t)\rangle =0\text{ and }\langle \xi_{i}(t)\xi_{i}(t^{\prime })\rangle =2\text{D}\delta (\text{t}-{\text{t}}^{\prime })$. *I*(*t*) is the pulse-like inputs with amplitude of Δ*I* and width of 1 *ms*.

**Table 1:** Parameters and rate functions of the HH neuron model.

C	membrane capacitance	1 μ*F*/*cm*^2^
*E*_*Na*_	Sodium reversal potential	50 mV
*E*_*K*_	Potassium reversal potential	−70 mV
*E*_*L*_	Leakage reversal potential	−54.4 mV
*g*_*Na*_	Maximal sodium conductance	120 mS/cm^2^
*g*_*K*_	Maximal potassium conductance	36 mS/cm^2^
*g*_*L*_	Leakage channel conductance	0.3 mS/cm^2^
α_*m*_		$\frac{0.1\left(\text{V}+40\right)}{1-{\text{e}}^{-1\left(\text{V}+40\right)/\text{10}}}$
β_*m*_		4e^−(V+65)/18^
α_*n*_		$\frac{0.01\left(\text{V}+55\right)}{1-{\text{e}}^{-\left(\text{V}+55\right)/80}}$
β_*n*_		0.125e^−(V+65)/10^
α_*h*_		0.07e^−(V+65)/20^
β_*h*_		$\frac{1}{1+{\text{e}}^{-\left(\text{V}+35\right)/10}}$

In this study, we defined the E/I cell ratio as the ratio of the number of excitatory neurons to the number of inhibitory neurons. For different E/I cell ratio, we first determined the number of the excitatory neurons, and chose them randomly from the N neurons in the network. The remaining neurons were considered as inhibitory neurons. The interneuron connections were set so that the excitatory neurons sent out only excitatory synapses and inhibitory neurons sent out only inhibitory synapses to each other neurons. Thus, for this all-to-all connected network, supposing there are *N*_*E*_ excitatory and *N*_*I*_ inhibitory neurons in the network (*N*_*I*_ = *N* − *N*_*E*_), each excitatory neuron receive presynaptic inputs from *N*_*E*_-1 excitatory and *N*_*I*_ inhibitory neurons; each inhibitory neuron receives presynaptic inputs from *N*_*E*_ excitatory and *N*_*I*_-1 inhibitory neurons (as demonstrated in Figure 1A with 3 excitatory and 2 inhibitory neurons). Therefore, $I_{Syn}^{i}\left(t\right)$ in Eq. (1), the synaptic currents of any one neuron received from other neuron is written as $I_{Syn}^{i}=\overset{N}{\underset{j=1,j\neq i}{\sum }}g_{Syn}^{j}\left(t\right)\left(V_{i}-E_{Syn}^{j}\right)$ (Wittmeier et al., 2008). In our simulation, the reversal potential of the excitatory synapse was set to 40 mV (i.e., $E_{Syn}^{j}=40mV$ if the j-th neuron is the excitatory neuron), whereas that of the inhibitory synapses was set to −94 mV, (i.e., $E_{Syn}^{j}=-94mV$ if the j-th neuron is the inhibitory neuron). The time-dependent changes in synaptic conductance was modeled by a single exponential with decay time constant of τ = 1 ms, the time-dependent conductance g_Syn_(t) of a synapse is defined as

(3) $${\text{g}}_{Syn}^{j}\text{(t)}=[{\text{g}}_{Syn}^{j}\;{\text{(t}}_{0}^{j}{\text{) +g}}^{j}]{\text{e}}^{(t_{0}^{j}-t)/\tau },$$

where ${\text{t}}_{0}^{j}$ is the time of the most recent firings of j-th neuron that have synaptic connections to i-th neuron. The synaptic conductance increase associated with one synaptic event was set to ${\text{g}}^{j}\text{=}{\text{g}}_{E}=0.012\text{ nS}$ if the j-th neuron is excitatory and ${\text{g}}^{j}\text{=}{\text{g}}_{I}\text{=0}\text{.1 nS}$ if the j-th neuron is inhibitory. To make sure the total synaptic current each neuron received from other neurons is in the acceptable range of HH model, the synaptic conductance in our simulation was 10 times smaller than that in Wittmeier et al. (2008). In the simulations, the number of neurons in the network was set to *N* = 250 if not specified. The differential equations were integrated with stochastic Euler method with time step of 0.1 ms.

### The measurement for mutual information

In the simulation, each neuron in the network receives the identical pulse input. The pulse strength Δ*I* are sampled from a uniform distribution which is in the range of [5.1, 6.9], with the discreted bin size of 0.1. This choice of input strength covers both the subthreshold and suprathreshold stimuli. The output of this network is discrete, i.e., R = {r|r = K; K = 0, 1, 2, …, N}, where K is the number of spikes in the network after the application of inputs. For each pulse strength, 1,000 times of trials are performed to calculate P(r|s), the probability distribution of output r for input s. Then the noise entropy is calculated with $H_{noise}=-\underset{s,r}{\sum }P\left(s\right)P\left(r|s\right)\log_{2}P\left(r|s\right)$, summarized over different inputs. The total entropy is calculated as $H_{total}=-\underset{r}{\sum }P\left(r\right)\log_{2}P\left(r\right)$ where $P\left(r\right)=\underset{s}{\sum }P\left(s\right)P\left(r|s\right)$ is the probability distribution of output r, without specifying the input s. Then the mutual information is calculated by subtracting the noise entropy from the total entropy

(4) $$\begin{array}{l} I_{M}=H_{total}-H_{noise}=-\sum_{r}P(r)\log_{2}P(r)\\ \;+\sum_{s,r}P(s)P(r|s)\log_{2}P(r|s). \end{array}$$

Therefore, the mutual information measures how much information is conveyed by the network with the firing patterns (the number of firings in the network which are supposed to carry information about the inputs). In principle, increasing the network size N results in larger repertoire of number of spikes, thus leads to higher information entropy (Zhang et al., 2015).

### Calculation of the network energy cost

In response to inputs, a network consumes energy during the action potential generation process and the synaptic transmission process. Two methods are commonly used to calculate the energy cost due to transmembrane voltage fluctuations, including action potentials. One method is to convert the Na^+^ current into the number of Na^+^ ions that enter into the cell body then estimate the amount of ATP required to extrude these ions from the cell (Attwell and Laughlin, 2001). The other method is to directly calculate the energy cost from the electrochemical energy function in the equivalent electrical circuit representing the HH neuron (Moujahid et al., 2011; Ju et al., 2016). However, to comply with our analytical solution from the bistable neuron model introduced below, we estimate the energy cost by counting the number of action potentials. If we assume each action potential costs one unit of energy, we can count the number of action potentials (N_spike_) generated in a response process, and the energy cost of a spike is then N_spike_. In this way, we ignore the energy cost due to subthreshold fluctuations. However, if the energy cost during a short period of neuronal activity is concerned, and within this period, neurons fire at high probability, this action potential counting method can yield a good estimation of the dependence of energy cost on the firing rate, or other factors that affect neuronal firing rate such as membrane area (Yu and Liu, 2014). Furthermore, as each spike causes N-1 synaptic events in this all-to-all connected network, the energy cost of synaptic events is proportional to α(N-1)N_**spike**_, assuming that one synaptic event costs α units of energy. The total energy cost of the network in response to the inputs is [1+α(N-1)]N_spike._ Attwell et al. estimated the energy expenditure on different components of excitatory signaling in the gray matter of rodent brain, they found that the energy expended on synapse (mainly due to releasing vesicles of glutamate) was just slightly less than the cost of the action potential (Attwell and Laughlin, 2001). However, recent study on human brain suggested a much higher ratio of energy cost in synapses to that in action potentials (Yu et al., 2017). Therefore, in the following context we set α = 2.5 by default, but in the next section we will show that our results hold for a wide range of α. In view of the fact that there is also an energy cost associated with ongoing spontaneous neuronal activities caused by noise, a constant fixed energy cost was also included in the total energy cost (Schreiber et al., 2002; Zhang et al., 2015) (See Discussion for details).

### The analytical bistable neuron model

Based on the analytical solution of our previous work (Yu and Liu, 2014; Yu et al., 2016), we also investigated the effects of the net synaptic current on the energy efficiency of a network composed of bistable neurons with E/I connections. The bistable neuron model is described by the following equation:

(5) $$\dot{v}=-U^{\prime }(v)+\Gamma (t),$$

where *v* is the membrane potential and *U* is a double well potential, defined as

(6) $$U=-\frac{a}{2}v^{2}+\frac{v^{4}}{4}$$

Note that *U* has two minima at $v_{s1}=-\sqrt{a}$, $v_{s2}=\sqrt{a}$ and a saddle point at *v*_μ_ = 0. In the following calculation, we set *a* = 1 by default. Γ(*t*) is the background Gaussian white noise, with

(7) $$<\Gamma (t)>=0;<\Gamma (t)\Gamma (t^{\prime })>=2D\delta (t-t^{\prime }),$$

where D is the noise intensity. We assume a neuron to be at its resting state when the particle is in the left well and excited when the particle crosses the barrier to the right well due to noise perturbation or signal stimulation.

Previously, we obtained the probability of finding a particle in the right well after a sufficiently long time, i.e., the probability that a pulse input signal is detected by the neuron (Yu and Liu, 2014),

(8) $$P_{c}(\Delta v)=\frac{1}{2}[1+erf(\frac{\Delta v}{\sqrt{2D/a}})],$$

where $\Delta v=v^{\prime }-v_{\mu }$ is the strength of the input pulse signal and *v*′ is the position of the particle after the pulse force is applied. Our previous work demonstrated that this solution captures well the noise-induced threshold fluctuation of single HH type neurons in response to pulse-like inputs (Yu and Liu, 2014).

We assume the existence of N bistable neurons that are all connected to one another with excitatory and inhibitory synapses. In this case, the firing probability of a bistable neuron in response to pulse inputs will also be affected by the synaptic currents it receives. Therefore, Equation 7 should also incorporate the effect of the synaptic current. However, a detailed description of this effect is difficult because the synaptic current is time-variant and depends on many variables, e.g., the number of firings in the network, the number of excitatory and inhibitory neurons, the coupling strength for excitatory and inhibitory synapses, and others. Here, we borrow a concept from mean field theory and consider the average effects of synaptic interaction for each neuron as a correction for the input Δ*v*. Let Δ represent the net synaptic current received by a bistable neuron from other neurons in the network, and assuming that this net synaptic current take effect instantly along with a pulse input Δ*v*, we can consider each bistable neuron in the network to actually receive a pulse input with strength Δ*v*+β · Δ, where β = κ · *D* represents the effect of the noise intensity on the synaptic current, *D* is the noise intensity, and κ is a scaling factor. Considering the above modification, the average pulse signal detection probability for a bistable neuron in the network can be written as

(9) $${\bar{P}}_{c}(\Delta v,\Delta )=\frac{1}{2}[1+erf(\frac{\Delta v+\kappa \Delta \;\cdot \;D}{\sqrt{2D/a}})].$$

With the above mean field assumption, we can treat the network as an array of *N* bistable neurons with pulse like inputs. Assume that the input strength is distributed uniformly over the interval [Δ*v*_min_, Δ*v*_max_], i.e., its probability distribution is as follows:

(10) $$q(\Delta v)=\frac{1}{\Delta v_{\max}-\Delta v_{\min}}=\frac{1}{\Delta s}.$$

Then, $\bar{v}=\frac{\Delta v_{\max}+\Delta v_{\min}}{2}$ is the mean value of the input strength. In the following calculation, we fix a distribution interval [−0.1 0.1]; thus, both subthreshold and suprathreshold inputs are involved. With the input ΔvϵS, the output of this network is discrete, i.e., *R* = *{r|r* = *K; K* = 0, 1, 2, …, *N*}, where K is the number of neurons excited after the inputs are applied. Following the method used in our previous work (Yu et al., 2016), we can calculate the mutual information of this network in response to pulse inputs, which is written as

(11) $$I_{M}(S;R)=\frac{1}{\Delta s}\sum_{K=0}^{N}\int_{\Delta v_{\min}}^{\Delta v_{\max}}q(r|\Delta v)\;\cdot \;\log_{2}\frac{q(r|\Delta v)}{q(r)}d(\Delta v).$$

The energy cost of this bistable neuronal network can be calculated as

(12) $$E_{total}=E_{0}+(1\text{+}\alpha (\text{N}-1))\int_{S}d(\Delta v)p(\Delta v)E_{\Delta v}(N,t),$$

where *E*_0_ is the fixed energy cost (see Discussion for details), and *E*_Δ*v*_(*N, t*) is the energy cost of the action potentials in response to input pulses with strength Δ*v*. Assuming each action potential costs one unit of energy, then $E_{\Delta v}\left(N,t\right)=N{\bar{P}}_{c}\left(\Delta v\right)$ if the inputs are applied at the beginning of this time interval, and it equals zero otherwise. Therefore, $\underset{S}{\int }d\left(\Delta v\right)p\left(\Delta v\right)E_{\Delta v}\left(N,t\right)$ is the average energy cost of action potentials in response to input pulses with distribution *p*(Δ*v*). Following the method used to calculate energy cost from synaptic activities, we introduce α as the ratio of the energy cost of one synaptic event to one action potential. Thus, $\alpha (\text{N-}1\text{)}\int_{S}d(\Delta v)p(\Delta v)E_{\Delta v}(N,t)$ is the energy cost of the total synaptic activity in response to pulse inputs.

## Results

### Network response to pulse inputs

Figure 1B shows sampled action potential traces and the corresponding synaptic currents of neurons in a network with E/I connections. When pulse-like inputs are applied, neurons with membrane potentials over the threshold are excited and send excitation/inhibition currents to the other neurons to which they are connected. Neurons may be excited by the excitatory synaptic current they receive. For example, a neuron that is not excited by inputs alone can be excited by the excitatory current it receives from other excited neurons (e.g., the membrane potential marked with a red line in Figure 1A; this phenomenon is also seen in the middle plot of Figure 1B, where the dashed red line marks the average synaptic currents received and the shadow marks the distribution of synaptic currents received from different neurons). Neurons that tend toward excitation (e.g., the membrane potential marked with a cyan line) may be inhibited by the inhibitory synaptic currents they receive (as shown at the bottom of Figure 1B, where the dashed cyan line marks the average synaptic currents received and the shadow marks the distribution of synaptic currents received from different neurons).

**Figure 2:**
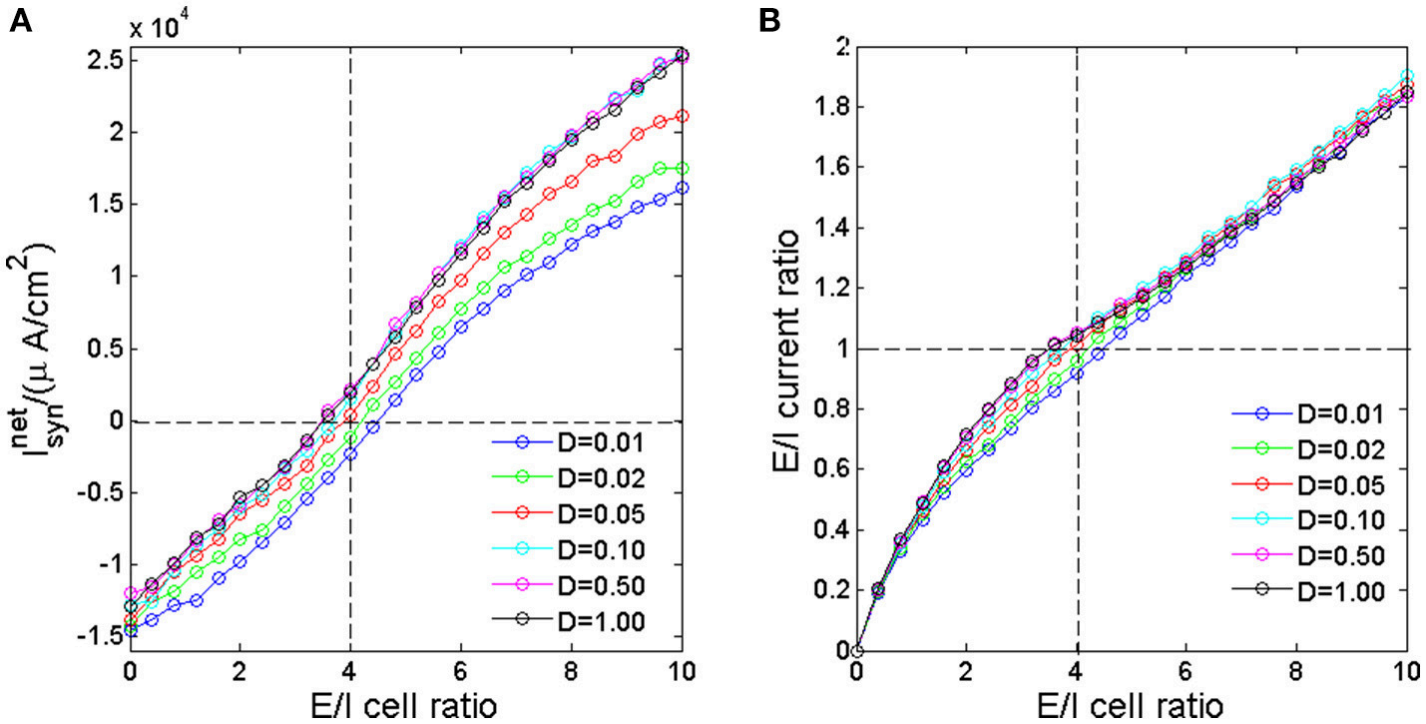
The total net synaptic current **(A)** and E/I current ratio **(B)** as a function of E/I cell ratio for different noise intensity. The horizontal dashed line is corresponding to the balanced excitatory and inhibitory synaptic currents. The vertical dashed line marks the E/I cell ratio of 80/20.

To investigate how the excitatory and inhibitory synaptic currents in the network vary as we adjust the ratio between the numbers of excitatory neurons to that of inhibitory neurons, we apply identical pulse inputs to each neuron at the same time; in each trial, the strength Δ*I* is sampled from a uniform distribution between [5.1, 6.9]. We then calculated the total net synaptic current in the network for different E/I cell ratio. The total net synaptic current $I_{syn}^{net}$ is calculated by integrating the net synaptic current at each time point in the interval of 0–8 ms after the application of input (Chen et al., 2008). It is seen form Figure 2A that the as E/I cell ratio increases, the total net synaptic current increases monotonically. When E/I cell ratio is low, the inhibitory synaptic currents generated after excitation of inhibitory neurons would dominate the network, resulting in negative total net synaptic current. Whereas, when E/I cell ratio is high, the excitatory synaptic currents would dominate the network, hence the total net synaptic current is positive. Around E/I cell ratio of 4 (vertical dashed line), the total net synaptic current is near zero (horizontal dashed line), which implies that the excitatory synaptic current is roughly balanced by their inhibitory counterpart. This result suggest that with the current setting of modeling parameters, through adjusting the ratio between the numbers of excitatory neurons to that of inhibitory neurons, the network could results in balanced synaptic current. The E/I current ratio, which is defined as the ratio of the total excitatory synaptic currents to the absolute value of total inhibitory synaptic currents in the 8 ms interval, increase monotonically as the E/I cell ratio increases (Figure 2B). And this ratio is approximately 1 when the E/I cell ratio is 4. It is also interesting to see that the E/I current ratio is nearly noise invariant when E/I cell ratio is small or large, but dispersed by noise with moderate E/I cell ratio around 4.

**Figure 3:**
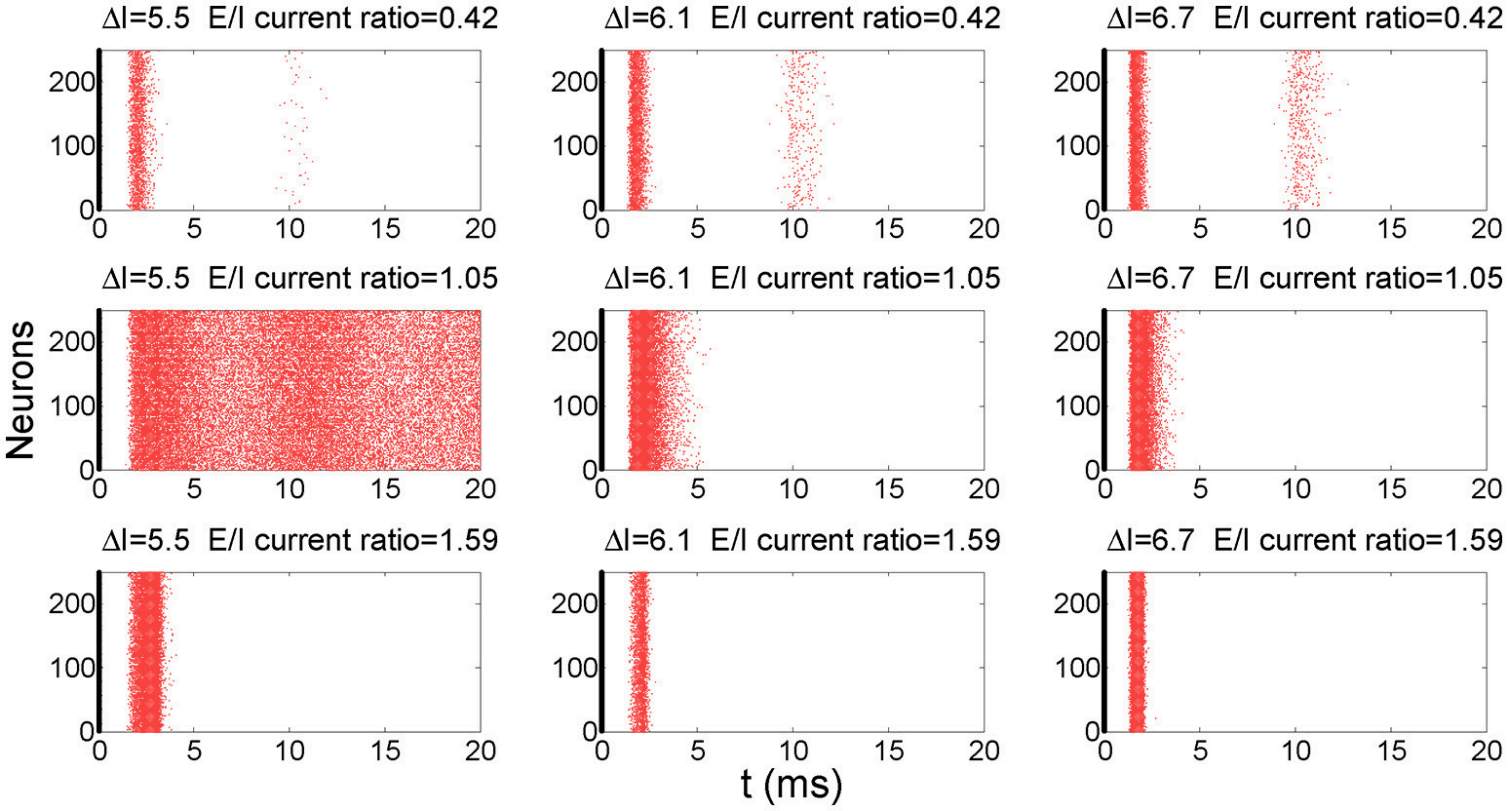
A post-stimulus raster plot of the firing activity in the network for different input strengths and E/I ratios. The thick lines at 0 ms indicate the time instant when the pulse inputs are applied. The raster plots of 100 trials are superimposed to better show the results. *D* = 0.1 in these simulations.

To demonstrate the response behavior of this network to above pulse current stimulation, we superimposed raster plots of 100 trials onto one plot to demonstrate how this network responds to pulse inputs. Figure 3 shows the superimposed post-stimulus raster plots of the global firing behavior of this network after the application of pulse inputs for different E/I current ratios and pulse strengths. In most cases, the network responds to input pulses with a burst of action potentials and then returns to a quiescent state. However, in some cases the noise would induce sustained spontaneous firings (e.g., Δ*I* = 5.5 and E/I current ratio = 1.05). In this study, we assumed the information in the input signals is carried by the first wave of the spikes and used a detection window of 8 *ms* after the application of inputs; the window is therefore large enough to include the first wave of firings but small enough to exclude sustained and ongoing spontaneous firings (Yu and Liu, 2014). In this arrangement, each neuron fires at most once within the detection window.

### Mutual information is maximized by the E/I current ratio

**Figure 4:**
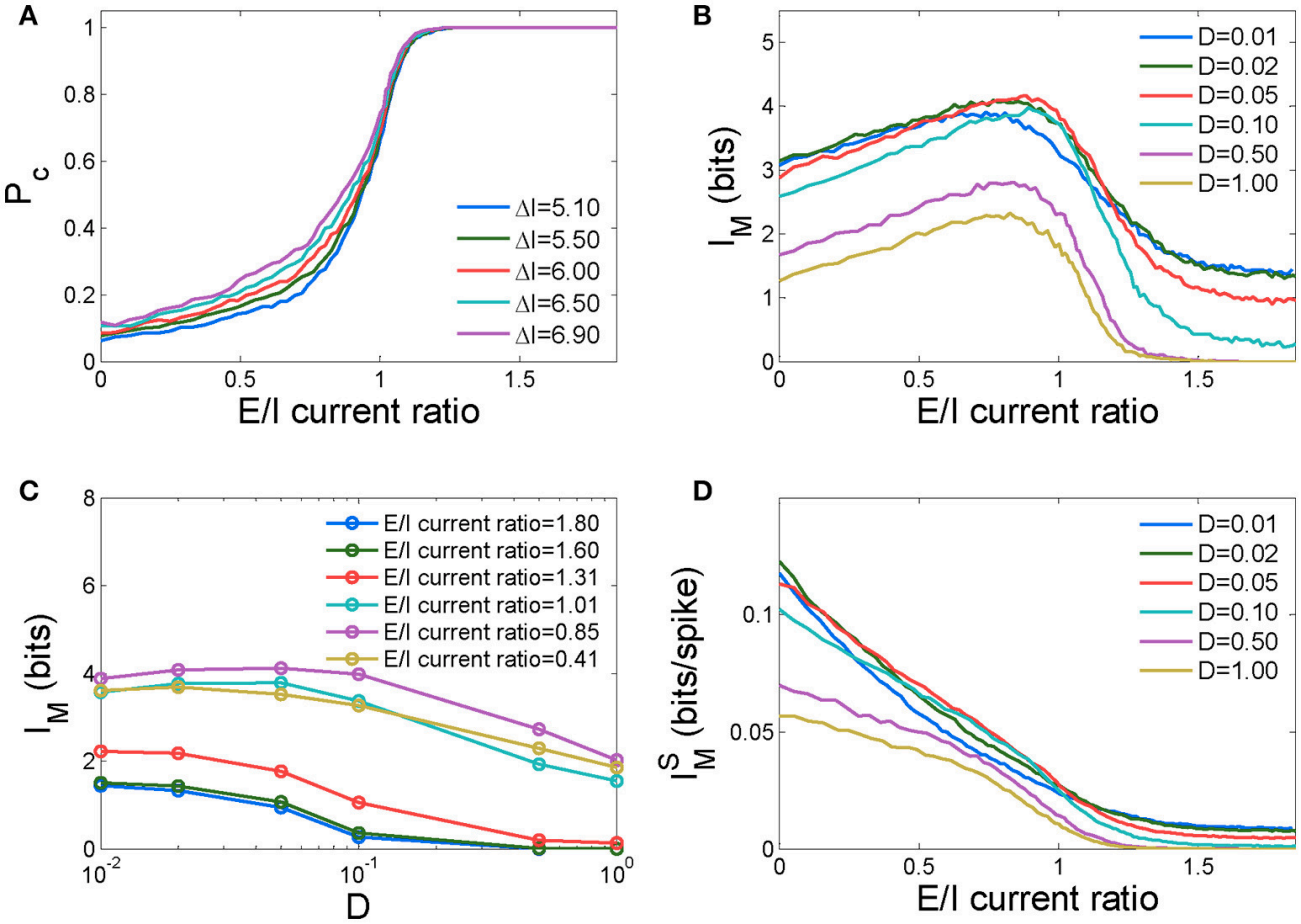
The dependence of the information capacity of the network on the ratio of the number of excitatory neurons to the number of inhibitory neurons in the network. **(A)** The pulse signal detection rate as a function of the E/I current ratio for different input pulse strengths. *D* = 1.0 in these calculations. **(B)** The mutual information as a function of the E/I current ratio for different noise intensities. **(C)** The mutual information as a function of the noise intensity for different E/I current ratios. **(D)** Mutual information per spike as a function of the E/I current ratio for different noise intensities. In the above calculations, the number of neurons *N* = 250.

The average detection rate per neuron in this neuronal network *P*_*C*_, which is defined as the total number of spikes in the detection window over the total pulse received by this network, increases as the E/I current ratio increases (Figure 4A). As the response time of a typical HH neuron is distributed within an interval of 0–8 *ms* after the application of input (Chen et al., 2008) and its synaptic transmission is not delayed, the inter-neuronal synaptic currents caused by early firings in the network will immediately affect later firings. If the E/I current ratio is low, the inhibition current is prominent, and later firings tend to be suppressed. In this case, larger inputs will lead to more early firings and thus a stronger inhibition current to suppress later firings. As a result, the difference in the detection rate for strong and weak pulses is trivial when the E/I current ratio is low. If the E/I current ratio is high, a weak signal causes fewer early firings than a strong pulse, but these early firings have a high probability of inducing other neurons to fire. The difference in the detection rate for strong and weak pulses again becomes trivial. Therefore, the difference in the detection rate for strong and weak pulses is largest at a moderate E/I current ratio.

In Figure 4B, we show the effect of the E/I current ratio on the mutual information in this network in response to pulse inputs at different noise intensities. As the E/I current ratio increases, the mutual information first increases and then decreases, and a maximum exists for medial values of the E/I current ratio (an E/I cell ratio between 2.3 and 3.5, which corresponds to 70–78% excitatory neurons and 30–22% inhibitory neurons in the network), which implies that mutual information could be maximized by the E/I cell ratio. High levels of noise tend to decrease mutual information globally and maximize mutual information at smaller E/I current ratios (the lines corresponding to *D* = 0.5 and *D* = 1.0 in Figure 4B). Lower levels of noise increase mutual information when the E/I current ratio is either low or high. However, when the E/I current ratio is in a moderate range, a moderate level of noise results in the most mutual information (the line corresponding to *D* = 0.05 in Figure 4B, or the line corresponding to E/I current ratio = 0.85 in Figure 4C). This phenomenon is known as “stochastic resonance,” a mechanism by which a nonlinear threshold system can enhance its signal to noise ratio when the noise intensity reaches an optimal level (Gammaitoni et al., 1998), and has been well studied in neural systems (Durand et al., 2013). The mutual information per spike, Ī_*M*_, which is defined as the ratio of the mutual information of the network *I*_*M*_ to the total number of spikes generated in the network in response to pulse signals, decreases monotonically as the E/I current ratio increases. This implies that the existence of fewer spikes in a network enables each spike to carry more information, which can be achieved by reducing the E/I current ratio of the network. For moderate E/I current ratios, the mutual information per spike is highest for moderately intense noise (e.g., *D* = 0.05 in Figure 4D).

**Figure 5:**
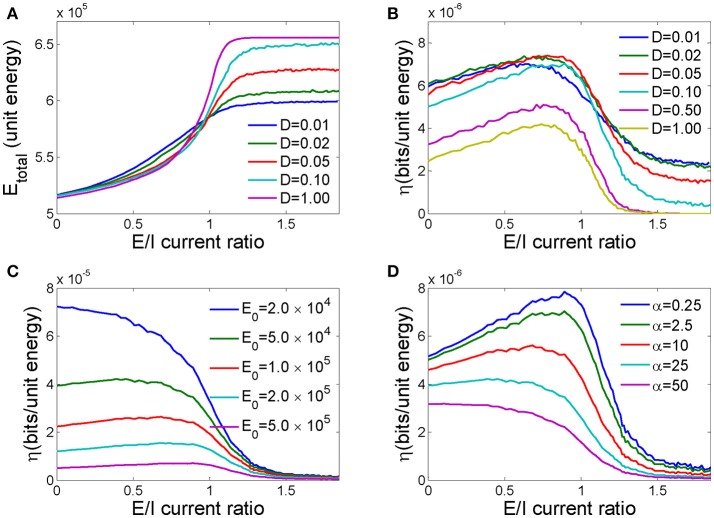
The effects of the E/I current ratio on the energy cost and energy efficiency of a neuronal network. **(A)** The total energy cost as a function of the E/I current ratio for different noise intensities. **(B)** The energy efficiency as a function of the E/I current ratio for different noise intensities. **(C)** The energy efficiency as a function of the E/I current ratio for different fixed energy costs. **(D)** The energy efficiency as a function of the E/I current ratio for different energy costs per synaptic event. In **C**,**D**, the noise intensity *D* = 0.1, and *N* = 250 in all simulations.

In Figure 5A, we plot the dependence of the total energy cost of the network undergoing the information processing activities described above on different E/I current ratios for different noise intensities. Because large E/I current ratios lead to more excited neurons in the network, the total energy cost generally increases as the E/I current ratio increases for different noise intensities. However, for large E/I current ratios, the noise intensity has a large effect on the energy cost, and high noise intensities cost more energy. In this range, high noise intensity will cause more neurons to fire in advance, and those firings will lead to more firings in other neurons through excitable connections. Therefore, a higher noise intensity increases firing probability and energy cost. However, when the E/I current ratio is low, lower noise intensities cost more energy.

Energy efficiency is defined as the ratio of mutual information to the energy cost in response to input pulses. We found that as the E/I current ratio of a network increases, the energy efficiency first increases and then decreases and peaks at a medially valued E/I current ratio (Figure 5B). This implies that there exists an optimal ratio of excitatory to inhibitory connections in the network at which the amount of information transmitted is maximized for a given unit energy cost. Because high noise intensity will sabotage information processing in the network, as demonstrated in Figure 4B, energy efficiency decreases as the noise intensity increases. However, there exists an optimal noise intensity that maximizes energy efficiency at a moderate E/I current ratio.

The vital role of fixed energy in the maximization of energy efficiency has been reported in several previous studies (Schreiber et al., 2002; Zhang et al., 2015). They found that the energy efficiency decreases monotonically as the system size increases if the fixed energy cost is not taken into consideration. Here in this paper, we found that the fixed energy cost is also important for the maximization of energy efficiency through the E/I current ratio. As demonstrated in Figure 5C, energy efficiency increases with decreasing fixed energy costs, and the optimal E/I current ratio for maximal energy efficiency trends toward smaller values. Finally, further decreasing the fixed energy cost eradicates the medial E/I current ratio peak, and energy efficiency decreases monotonically with an increasing in E/I ratio. Therefore, to maximize energy efficiency, the fixed energy cost should be approximately the same order of magnitude as the energy cost of information processing.

We further investigated how the scaling factor α, which represents the energy cost of a synaptic event with respect to the energy cost of one action potential, influences the energy efficiency of the network. As shown in Figure 5D, as α increases from 0.25 to 50, the energy efficiency decreases because synaptic activity requires more energy to transmit the same amount of information, and the optimal E/I current ratio for maximal energy efficiency decreases. Too large a value of α erases these peaks, resulting in a monotonic dependence of energy efficiency on the E/I current ratio.

### Maximized mutual information and energy efficiency as revealed by a simple bistable neuron model

**Figure 6:**
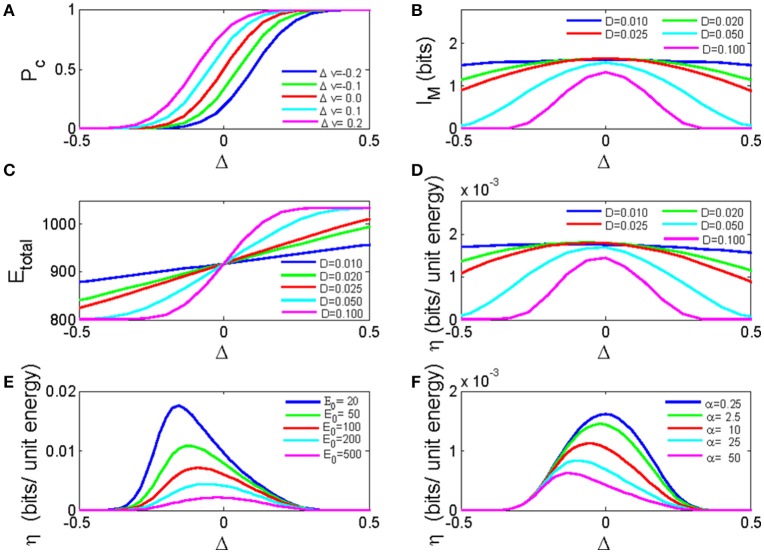
The analytic solution for a bistable neuron network. **(A)** The dependence of the pulse signal detection rate on the net synaptic current for different input signal strengths. *D* = 0.1; **(B)** The mutual information of a bistable neuron network as a function of the net synaptic current for different noise intensities. **(C)** The total energy cost of a neuronal network as a function of the net synaptic current for different noise intensities. **(D)** The energy efficiency as a function of the net synaptic current for different noise intensities E-0 = 24. **(E)** The energy efficiency as a function of the net synaptic current for different fixed energy costs. **(F)** The energy efficiency as a function of the net synaptic current for different energy costs per synaptic event. In these calculations, *N* = 10, and Δ*v*_min_ = −0.5. Δ*v*_max_ = 0.5 if not specified.

In the previous section, we have obtained the analytical solution for the network dynamics (Equation 9), information transmission (Equation 11), as well as the energy cost (Equation 12) of the bistable neuron network as a function of net synaptic current. Figure 6A demonstrated the dependence of pulse signal detection rate on the net synaptic current for different input signal strengths. If the E/I ratio is low, inhibitory synaptic currents will dominate the network, resulting in a small and negative Δ that decreases the probability of firings in response to signals. If the E/I ratio is high, excitatory synaptic currents dominate the network, resulting in a large and positive Δ that increases the firing probability. Comparing Figure 6A with Figure 4A, we find that Equation 9 captures the general properties for the dependence of the response probability on the E/I ratio, especially for neuronal responses to signals with different strengths, which are most easily discriminated at moderate E/I ratios, i.e., around Δ = 0.

Figure 6B plots the mutual information as a function of Δ for different noise intensities. When the excitatory and inhibitory synaptic currents are balanced (Δ = 0), the mutual information is maximized, and smaller noise intensities yield higher mutual information.

As shown in Figure 6C, when the net synaptic current is larger than zero, the energy cost increases as the net synaptic current increases, and higher noise intensities result in higher energy costs. However, when the net synaptic current is <0, smaller noise intensities result in higher energy costs. This result is in accordance with the simulation results. However, in the simulation, the energy cost curves for different noise intensities tend to diverge when E/I is large, and these curves are compressed when E/I is small (as shown previously in Figure 5A). Because of the over-simplified consideration of inter-neuronal connections in the simulation, our analytical result fails to replicate these behaviors. Anyway, the solution for energy efficiency (η = *I*_*M*_/*E*_*total*_) still exhibits maxima, and there exists an optimal noise intensity that maximizes energy efficiency around a net zero synaptic current (Figure 6D). Figures 6E,F show that the energy efficiency decreases as the fixed energy cost or the energy cost for synaptic activity increases. The optimal net synaptic current for maximal energy efficiency moves to the left with an increased fixed energy cost and moves to the right with an increased synaptic energy cost. Thus, our analytical solution is consistent with the simulation results described above.

## Discussion

The E/I balance is an important feature of a cortical neuronal network that influences many aspects of cortical neurons (Bernander et al., 1991; Paré et al., 1998; Shadlen and Newsome, 1998; Destexhe et al., 2003; Wehr and Zador, 2003; Mittmann et al., 2005; Wilent and Contreras, 2005; Wolfart et al., 2005; Rudolph et al., 2007), including their information processing ability and energy consumption (Sengupta et al., 2013). In this study, we investigated the dependence of the information transmission and energy efficiency of a neuronal network on the balance of excitatory and inhibitory synaptic currents through both computational simulation of classical HH neurons and analytic solution of bistable neurons with a mean-field approximation. Our results suggest that the excitatory and inhibitory synaptic currents can be canceled with each other (or well balanced) by an optimal E/I cell ratio, and the E/I synaptic current ratio of 1. We further demonstrated that with this balanced E/I synaptic current, the information transmission and energy efficiency of the neuronal network could be maximized. We also identified that the fixed energy cost is necessary for energy efficiency to be maximized, and it also influence the optimal E/I synaptic current ratio which maximize the energy efficiency.

Our results are in accordance with previous studies of information capacity and transmission in E/I balanced neuronal networks. Previous studies on the E/I current balances of the cortices of rats and monkeys have shown that balanced excitation and inhibition leads to neural avalanches whose sizes follow a power law distribution, suggesting that neural systems are poised around a critical point (Beggs and Timme, 2012; Poil et al., 2012; Hesse and Gross, 2014). Many studies have found advantages to neuronal networks for organizing around this critical point, such as maximized information storage (Haldeman and Beggs, 2005), maximized dynamic range (Shew et al., 2009), and maximized information transmission (Shew et al., 2011). As argued in Shew et al. (2011), a large E/I synaptic current ratio leads to a super-critical state in which neurons are highly activated and spikes among neurons are highly correlated. However, a small E/I synaptic current ratio leads to a sub-critical state in which the overall level of neural activity decreases and the spikes among neurons are random and uncorrelated. Highly correlated spikes reduce entropy in the former case, and reduced correlation increases entropy in the latter case, but this increase is counteracted by the concurrent decrease in total information, resulting in maximal information transmission at a moderate E/I ratio. Our results also demonstrate that through stochastic resonance phenomenon in which the optimal noise intensity maximizes the information transmission of a nonlinear threshold system, the noise could enhance the information transmission in the network (Figures 4B–D, 6B).

In previous decades, many studies have shown that to process information in an energy efficient manner, neuronal systems optimize their morphological and physiological parameters, e.g., ion channel kinetics, body temperature, number of channels on single neurons and number of neurons in neuronal network, and the intra- and intra-region wiring of the cortex (Chklovskii and Koulakov, 2004; Alle et al., 2009; Schmidt-Hieber and Bischofberger, 2010; Yu et al., 2012, 2016). Sengupta et al. assessed the impact of balanced synaptic currents on information coding and energy consumption in a single HH-type neuron driven by one of three synaptic input regimes: excitatory inputs only, balanced synaptic conductance, or balanced synaptic currents. They found that spikes evoked by balanced synaptic currents are more informative and energy efficient (Sengupta et al., 2013). Recent work also revealed that the cost-efficient information capacity with minimal spike rate can be achieved in the regime of moderate synchrony, where the irregular firing, synchronized oscillations and neuronal avalanches can be observed simultaneously (Yang et al., 2017). Here, we showed that by tuning the E/I cell ratio in a network, the balanced excitatory and inhibitory current in a neuronal network enables the highest level of energy efficient information transmission. Therefore, our results, along with those of other studies, demonstrate the possibility that neural systems may optimize their morphological and physiological parameters to be energy efficient. However, though balanced excitation-inhibition network often leads to critical-state dynamics (Poil et al., 2012; Yang et al., 2017), we focused on the accurate ratio of E/I synaptic current and its impact on coding and energy efficiency after the network is evoked by the external inputs. Our current work did not take into account of the nontrivial dynamics patterns and its interaction with firing rates, which is left for our future study.

For energy efficiency to peak at the optimal E/I ratio, a large fraction of the fixed energy cost must be included in the total energy cost (Figure 5C). The dependence of the maximization of energy efficiency on the fixed energy cost has been reported in several studies. For example, both in a single neuron with graded potentials and a neuronal population, the maximization of energy efficiency by the number of ion channels or the number of neurons requires the inclusion of a fixed energy cost (Schreiber et al., 2002; Zhang et al., 2015). Here, we argue that this fixed energy cost could be assigned to the cost of generating spontaneous firings and consequent synaptic activity due to noise perturbation. In our calculation, the costs directly related to signal processing (the energy consumed by action potentials invoked by input signals and synaptic transmission) are explicitly calculated. However, spontaneous firing is an ongoing process that continually costs energy even without input signals. Normally, this energy cost is a constant within a unit time interval, assuming the spontaneous firing rate is a constant. Therefore, a larger fixed energy cost can be considered a longer interval within which no inputs are applied. Therefore, we speculate that to maximize energy efficiency, the signal input rate must be below a certain threshold so that a sufficient fraction of the fixed energy cost can be accounted for in the total energy cost. The energy cost of synaptic events greatly affects the energy efficiency of a network. In our calculations, we assume that synaptic transmission by a neuron costs α = 2.5 times the energy cost for action potentials at a 1 Hz firing rate (Howarth et al., 2012). And our results also show that the maximal energy efficiency holds even for a 10-fold increase or decrease of α, suggesting that for real neural systems the energy cost of a synaptic event is within the range that maximizes energy efficiency.

In this study, we expanded our previous solution of the response function for a bistable neuron from a single isolated neuron to neurons with excitatory and inhibitory synaptic connections by adding a modification term to represent the effects of a net synaptic current and noise on the firing probability of a neuron to pulse inputs. This mean field approximation is a simplification of the complex interactions between neurons, although in some cases, it fails to replicate the exact behavior of our simulation results (e.g., the dependence of the energy cost on the E/I ratio, comparing Figures 5A, 6C), it captures the essential behavior of the mutual information and energy efficiency with respect to the E/I ratio. Therefore, we expect that a more explicit form of this interaction term would lead to a more accurate description of the average response of the neurons in a network and would improve our understanding of the dynamics of neuronal networks with excitatory and inhibitory connections. This will be a direction for our future research.

It is noticed that previous work suggested that through tuning the ratio of excitatory to inhibitory synaptic current intensity, the network could be well balanced to maximize the energy efficiency (Shew et al., 2011; Yang et al., 2017). Whereas, our simulation work suggested that the balanced synaptic current and the most efficient information processing can also be achieved through tuning the E/I cell ratio with fixed synaptic coupling strength. This result suggests that a certain ratio (e.g., 80/20 ratio) of excitatory to inhibitory neurons in cortex is possibly an evolved optimal solution toward energy efficiency in functions. Since both changing E/I cell ratio and E/I synaptic ratio can result in balanced synaptic current which enable the network to be energy efficiency, which one does the nature prefer to chose if maximization of energy efficiency is a necessary for evolution? We have to point out the limitation of our work. First, information is not only carried by the firing rate, but also in the spike-timing interval as well as the population correlations (Panzeri et al., 2015), while we only consider the firing rate coding here. Second, in real cortex, there are multiplex network configurations, and most of them are sparsely connected. However, our analysis and results are derived from a fully recurrent connected network configuration, which might only provide a linear-style understanding on the principles of the cortical network organization. In this work, we used a uniform recurrent network structure that neurons are all-to-all connected. Recent work demonstrated that structural heterogeneity in cortical network could undermine the balanced state, while homeostatic synaptic plasticity can recover the balance of network excitation-inhibition (Landau et al., 2016). Therefore, more studies are needed to test if the conclusion obtained here still hold in the biologically more realistic case.

## Conclusion

In this study, we examined the energy efficiency of the information coding process of a neuronal array network composed of Hodgkin-Huxley neurons interconnected with excitation/inhibition synaptic couplings. We found that the E/I current ratio, which is defined as the ratio between the excitatory and inhibitory synaptic currents, exists an optimal range where both the information transmission and the energy efficiency of the network reach the global maximal level. These results are further confirmed by an analytical solution for a bistable neuron in which interconnection between neurons is approximated with a mean-field approach. The novel result obtained here reveals a general rule of energetics related to population coding that there exists an optimal excitation/inhibition ratio in the cortex necessary for maximal information transmission with minimal energy cost. These results reflect general mechanisms for sensory coding processes that may provide insight into energy efficient neural communication and coding.

## Author contributions

YY, LY, ZS, and CW: designed research; LY, ZS, and CW: performed research; LY, YY, and ZS: wrote the paper. All authors reviewed the manuscript.

### Conflict of interest statement

The authors declare that the research was conducted in the absence of any commercial or financial relationships that could be construed as a potential conflict of interest.
